# A Cytochrome b561 with Ferric Reductase Activity from the Parasitic Blood Fluke, *Schistosoma japonicum*

**DOI:** 10.1371/journal.pntd.0000884

**Published:** 2010-11-16

**Authors:** Amber Glanfield, Donald P. McManus, Danielle J. Smyth, Erica M. Lovas, Alex Loukas, Geoffrey N. Gobert, Malcolm K. Jones

**Affiliations:** 1 Queensland Institute of Medical Research, Herston, Queensland, Australia; 2 School of Population Health, The University of Queensland, Brisbane, Queensland, Australia; 3 School of Veterinary Sciences, The University of Queensland, Brisbane, Queensland, Australia; 4 Queensland Tropical Health Alliance, James Cook University, Cairns, Queensland, Australia; René Rachou Research Center, Brazil

## Abstract

**Background:**

Iron has an integral role in numerous cellular reactions and is required by virtually all organisms. In physiological conditions, iron is abundant in a largely insoluble ferric state. Ferric reductases are an essential component of iron uptake by cells, reducing iron to the soluble ferrous form. Cytochromes b561 (cyts-b561) are a family of ascorbate reducing transmembrane proteins found in most eukaryotic cells. The identification of the ferric reductase duodenal cytochrome b (dcytb) and recent observations that other cyts-b561 may be involved in iron metabolism have opened novel perspectives for elucidating their physiological function.

**Methodology/Principal Findings:**

Here we have identified a new member of the cytochrome b561 (Sjcytb561) family in the pathogenic blood fluke *Schistosoma japonicum* that localises to the outer surface of this parasitic trematode. Heterologous expression of recombinant Sjcyt-b561 in a *Saccharomyces cerevisiae* mutant strain that lacks plasma membrane ferrireductase activity demonstrated that the molecule could rescue ferric reductase activity in the yeast.

**Significance/Conclusions:**

This finding of a new member of the cytochrome b561 family further supports the notion that a ferric reductase function is likely for other members of this protein family. Additionally, the localisation of Sjcytb561 in the surface epithelium of these blood-dwelling schistosomes contributes further to our knowledge concerning nutrient acquisition in these parasites and may provide novel targets for therapeutic intervention.

## Author Summary

Parasites acquire their food from their hosts, either by feeding directly on tissues of the host, or by competing for ingested food. Adult schistosomes live within the vasculature of humans and rely on the blood cells and plasma they ingest and dissolved solutes they derive across their body surface, the tegument, for their nutrition. Schistosomes require host trace elements, notably iron, which is used as a co-factor in many biological reactions. Iron is especially important for schistosomes, for it has a significant role in egg formation and embryogenesis. In human tissues, iron predominates in the trivalent (ferric) form; however, it is the divalent (ferrous) form that is used as an essential co-factor for multiple biomolecules and enzymes. In order to be acquired from the host environment, the valency of iron must be modified to render it suitable for transport across the parasite membrane. This paper describes the molecular characterisation of a schistosome molecule that is crucial for bringing about this change in iron. *Schistosoma japonicum* Cytb561 is the first ferric reductase characterised in any parasitic helminth and emphasises the importance of iron, and other divalent cations, in these organisms.

## Introduction

Iron is an essential co-factor of many biological processes in nearly all organisms, and serves as a major strengthening and stabilizing metal in invertebrates [1]. Although iron is the second most abundant element on Earth and the fourth most abundant element in the crust, it exists in inorganic form, most often as insoluble trivalent ferric hydroxide or ferric oxide salts, forms that are not readily bio-available to organisms [2]. In mammalian tissues, iron is predominantly stored or transported by an array of molecules, including organic chelates, the serum transporter transferrin, or in the cytoplasmic storage complex ferritin, in its ferric form [3], [4]. However, it is in the divalent, or ferrous, state that iron participates as a co-factor in biological processes. Accordingly, it is necessary for cells to be able to reduce and solubilise iron in order to use it for a variety of cellular functions.

Early studies showed that eukaryotes acquire iron from their environment more readily as a ferrous ion. In yeast, chelators of ferric iron do not inhibit transmembrane iron transport and uptake, whereas ferrous chelators do [2], [5]. This biological characteristic has led to the identification and functional characterization of many ferric reductases, molecules able to convert ferric to ferrous iron, from a wide range of organisms, most notably the well studied FRE family of metalloreductases of yeast and the ferric reductase oxidase (FRO) proteins of plants [6], [7], [8], [9], [10], [11], [12], [13]. In mammals, the two major ferric reductase families that have been characterised include the cytochrome b561 homologues, among which the duodenal cytochrome b (Dcytb) is known most prominently, and the Steap family of metalloreductases [4], [10], [14], [15].

Schistosomes, platyhelminth parasites of humans, and other mammals, are a major source of human morbidity in many developing countries in tropical areas [16]. Adult schistosomes live within the vasculature of their human hosts and feed predominantly on erythrocytes, which are ingested, lysed and digested in a primitive gut, the gastrodermis. Females, in particular, have high metabolic requirements for iron, which is stored in abundance in vitelline, or egg shell precursor cells, to be used subsequently for embryogenesis, where it is thought to assist in stabilization of protein cross-links in the tanned eggshells [17], [18]. In addition to this choriogenic requirement, schistosomes also depend on iron for early establishment and growth in the human host [19]. In view of the importance of iron in the development of schistosomes, there has been growing interest in understanding how they acquire iron in their parasitic environment as a means to discovering new drug and vaccine targets for their control [20].

The surface of schistosomes consists of a syncytial anucleate layer, known as the tegument. This cytoplasmic layer serves as a nutritive and protective surface, and acts in immune evasion and, possibly, signalling for parasite development [21], [22]. A surface-associated pathway for iron absorption has been postulated for schistosomes [19], [20]. This hypothesis is supported by recent functional analysis of the membrane spanning divalent metal transporter (DMT1) family of proteins in *Schistosoma mansoni* [23]. The schistosome DMT1s, which show a tegumentary distribution by immunofluorescence microscopy, have been demonstrated to transport ferrous iron when expressed heterologously in yeast [23]. The presence of a membrane-spanning protein in schistosomes that transports ferrous iron into cells thus suggests that these blood-dwelling parasites must, of necessity, express molecules with ferric reductase activity [20]. To date, however, no functional ferric reductase has been characterised in schistosomes, or any other helminth.

One family of proteins recently identified as having ferric reductase capability are the cytochromes b561. These are intrinsic membrane proteins, containing two heme molecules, and are considered to be reducible by ascorbate [24]. Initially thought as only electron transporters [25], b561 family members were shown to have ferric reductase capacity following functional characterization of a mammalian duodenal cytb561 (dcytb) [26], [27], [28], [29]. Expressed sequence tags of putative cytb561 family members have been shown to be present in the genomes of *S. japonicum* [15], [30], [31] and *S. mansoni* [32],[33]. Here we describe the identification and molecular characterisation of a protein of the cytb561 family (Sjcytb561) from *S. japonicum* and demonstrate that it has functional ferric reductase activity and occurs in the tegument of these blood-dwelling parasites.

## Materials and Methods

The use of mice in this study was approved under Project P288 by the Animal Ethics Committee of the Queensland Institute of Medical Research.

### Isolation of RNA from schistosomes

*Oncomelania hupensis hupensis* snails, infected with a Chinese mainland strain of *S. japonicum* (Anhui Province population), were kindly provided by the National Institute of Parasitic Diseases-CDC, Shanghai, China. Adult worms were perfused from BALB/c mice 4 or 7 weeks after cutaneous infection with 30 *S. japonicum* cercariae. Eggs were collected following their extraction from the livers of infected mice by digestion of liver matrix by collagenase B and differential centrifugation using Percoll [34]. Miracidia were hatched from eggs by exposure to freshwater. The human-invasive cercariae were harvested from infected *O. h. hupensis* snails that were shed by exposure to direct light for approximately 2 h. Lung schistosomula were isolated from infected mice using a previously published procedure [35]. Approximately 1,000 cercariae were pooled from several infected *O. h. hupensis* snails, and used to challenge female BALB/c mice. Three days later the lungs were removed, minced with a razor blade and incubated in RPMI at 37°C for 3 h on a shaker. The lung tissue solution was sieved and schistosomula removed using a fine tipped glass pipette. Total RNA was obtained from the five different life stages by homogenizing parasite tissue in Trizol (Invitrogen) according to the manufacturer's instructions.

### Full length cDNA synthesis by RACE PCR

Expressed sequence tags (EST) encoding schistosome cytb561 were first identified by BLAST searches of the *S. mansoni* and *S. japonicum* EST databases. The GeneRacer kit (Invitrogen) was used for full length Rapid Amplification of 5′ and 3′ ends (RACE). Total RNA from adult worms was used and cDNA synthesis was carried out following the GeneRacer protocol. Numerous primers were designed based on ESTs and used in conjunction with proprietary GeneRacer 5′ and 3′ universal primers to amplify the full-length *Sjcytb561* sequence. This cDNA was amplified using Platinum taq DNA polymerase High Fidelity (Invitrogen), with the following cycling parameters: initiation at 96°C for 5 min, and 35 cycles of denaturation (94°C for 1 min), annealing (50°C for 1 min) and extension (68°C for 2 min).

### Stage-specific expression of *Sjcdytb561*

Transcriptional patterns for the different *S. japonicum* life- stages were evaluated using real time PCR. Primers were designed to amplify sequences for *Sjcytb561* (forward: 5′-TGGACCAATGCAAACACAGT-3′; reverse: 5′-TGATTCCCAGGACACCAAAT-3′) and a region of *S. japonicum* NADH ubiquinone reductase (forward: 5′-CGAGGACCTAACAGCAGAGG-3′; reverse: 5′-TCCGAACGAACTTTGAATCC-3′), used as a constitutively expressed control as previously described [35], [36]. All cDNA samples synthesised from total RNA were adjusted to 5 ng/µL, and quantified using a NanoDrop ND-1000 spectrophotometer (Thermo Scientific). Five µL aliquots of cDNA were then combined with 10 µL of SYBER Green (Applied Biosystems), 3 µL of water and 2 µL (5 pmol) of each forward and reverse primer in a Gene-Disc 100 ring (Qiagen) using a CAS-1200 automated PCR setup robot (Qiagen). Cycling conditions were: 10 minutes at 93°C followed by 40 cycles of 93°C for 20 seconds, 60°C for 15 seconds and 72°C for 15 seconds. All reactions were performed in a Rotor-Gene (3000) thermal cycler (Qiagen) and the data analysed using Rotor Gene 6 software (Qiagen). A standard curve was created with a mixed template (contained 50 µL from each separate template) and the mean copies per reaction values were calculated from the mean of 3 normalised CT (cycle threshold) values. All PCR experiments were conducted in triplicate.

### Genomic sequence and phylogenetic analysis of *Sjcytb561*

The full-length cDNA of *Sjcytb561* was cloned into the pCR4-TOPO vector (Invitrogen) and chemically transformed in *E. coli* TOP10 cells (Invitrogen) according to the manufacturer's guidelines. Colonies were screened by colony PCR using a combination of vector and insert primers. Plasmid mini-preps were prepared (Qiagen) and the sequence verified from plasmids using M13F and M13R vector primers and Big Dye sequencing chemistry (ABI) on an ABI automated DNA sequencer. The consensus sequence was compared with those already deposited in GenBank and the dbEST databases using the BLAST algorithm on the NCBI server. Topological data were obtained using the TMPRED server (http://www.ch.embnet.org/software/TMPRED_form.html) and the SignalP server (http://www.cbs.dtu.dk/services/SignalP/) was used to predict the presence of signal peptide cleavage sites. Multiple sequences were aligned using the Clustal W 1.8 algorithm on the EBI server (http://www.ebi.ac.uk/Tools/clustalw2/index.html). A phylogenetic tree was constructed using MEGAv3.0 software for Molecular Evolutionary Genetics Anaylsis [37] to compare *S. japonicum* and *S. mansoni* DMT1 sequences with homologous protein sequences identified from the public databases by BLAST searches. A minimum evolution phylogenetic tree was constructed using the JTT substitution model, with uniform rates among sites being assumed. The dataset was bootstrapped 1000 times, with the resulting values shown on branches of the midpoint-rooted tree. The Sjcytb561 cDNA sequence was used to interrogate the recently published genomic dataset for *S. japonicum* [31] to gain insight into genomic organization of the molecule. Alignment between the cDNA and genomic sequences was performed using SIM 4 [38], (http://pbil.univ-lyon1.fr/), to identify exons and introns in the gene sequence.

### Generation of anti-Sjcytb561 serum

Rabbit antiserum was raised against a synthetic peptide (CISGITEKNFFSKNY) corresponding to a region of the third extracellular loop of Sjcytb561. The region was chosen due to its extracellular location, its predicted immunogenicity (Invitrogen PeptideSelect; http://peptideselect.invitrogen.com/peptide/) and its lack of homology to mammalian b561 homologues, thereby reducing cross-reactivity with host tissues in the immunocytochemistry studies. The peptide was commercially synthesised and conjugated to Keyhole Limpet Hemocyanin (KLH) as carrier protein (Sigma). The Institute of Medical and Veterinary Science (IMVS), Adelaide, South Australia prepared the antiserum in a New Zealand White rabbit against the cytb561-KLH peptide using a protocol of 4 immunisations of 2 mg at weekly intervals. Freund's Complete Adjuvant (FCA) was used for the first immunisation and Freund's Incomplete Adjuvant (FIA) for the subsequent three immunisations. Serum was collected 2 weeks after the fourth immunisation and control, pre-immune serum was collected prior to commencement of the immunisation schedule.

### Purification of Sjcytb561-peptide reactive antibodies

Cyanogen bromide-activated Sepharose 4B (GE Healthcare), coupled with KLH, was used to deplete the anti-b561-KLH peptide antiserum of KLH immunoreactive antibodies. Serum was diluted at 1∶1 with PBS pH 7.4, added to the Sepharose 4B and rotated end over end, at 4°C over night, to allow efficient binding of KLH-specific antibodies. The flow through was collected to obtain purified anti-cytb561 peptide antibodies. Antibodies showing specific immunoreactivity against KLH were eluted from the column using 100 mM glycine, pH 2.5, then equilibrated by adding 1/10 volume of Tris-HCl, pH 8.0. Specificity of the anti-cytb561 and KLH reactive antibodies was confirmed by dot and Western blot analysis of a detergent-solubilized membrane extracts of *S. japonicum* (see below) and KLH solution.

### Western blot analysis of Sjcytb561

A protein extract from the tegument was prepared following methods of van Balkom and colleagues [39]. Five µg of adult parasite extracts (either tegument or ‘stripped’) and commercial KLH (Sigma) were separated by gel electrophoresis on a 12% (w/v) SDS-PAGE gel at 200 V for 1 hour at room temperature (RT) and then transferred to nitrocellulose membrane at 200 mA for 1 hour at RT. The membrane was blocked for 1 hour at RT in 6% (v/v) skim milk powder diluted in PBS-T. All washes were carried out in PBS-T. The membrane was incubated with primary SjCytb561 antiserum a diluted at 1∶100,000. A pre-immune serum was used at equivalent dilutions for the negative control. In addition anti-KLH specific antibodies were used as a control. After incubation with the primary serum, the membrane was washed for 15 minutes in PBS-T and incubated with horseradish peroxidase-conjugated goat anti-rabbit IgG (1∶2000, BioRad) for 1 hour at RT. The membrane was washed a further 3 times (5 minutes each) in PBS-T and then developed with chemiluminescence (ECL Plus, Amersham).

### Immunolocalisation of Sjcytb561

Freshly perfused, unfixed adult *S. japonicum* worms were embedded in Tissue-Tek Optimal Cutting Temperature (OCT) compound (ProSciTech) and snap-frozen on dry ice. The block was then cryostat-sectioned at a thickness of 7.0 µm and mounted onto Superfrost slides. Sections were labeled using an indirect immunofluorescence labelling protocol, whereby sections were initially blocked for 1 hour at room temperature (RT) in 2% (w/v) skim milk powder in PBS-Tween 20 (0.5%) (PBS-T), washed 3 times (5 minutes each) with PBS-T, and incubated with Sjcytb561 antisera at a 1∶1000 dilution. Sections were also incubated with pre-immune serum acting as a negative control. After washing, sections were incubated with goat anti-rabbit IgG secondary antibody conjugated to Cy3 fluorophore (Jackson ImmunoResearch Laboratories) at a 1∶500 dilution. After 3 further washes with PBS-T, slides were air-dried briefly and sections were mounted in Vectorshield mounting medium (Vector Labs) that contained a 4′, 6-diamidino-2-phenylindole (DAPI) counterstain. Sections were examined and photographed using a Leica DM IRB fluorescence microscope equipped with a Leica DC 500 digital camera.

### Immuno-electron microscopy

Freshly perfused adult worms were fixed in 4% (v/v) paraformaldehyde and 0.1% glutaraldehyde in 0.1 M phosphate buffer for 1 hour. Worms were then dehydrated in ethanol and infiltrated and embedded in LR White resin (ProSciTech) blocks. The blocks were sectioned on an ultramicrotome (Leica EM UC6). Sections were washed for 3×5 minutes in PBS, then blocked for 15 minutes in a blocking buffer consisting of 200 µL 10% (w/v) bovine serum albumin (BSA), 200 µL 10% (w/v) fibroblast surface glycoprotein (FSG), 1 mL 200 mM glycine, 2 mL phosphate buffered saline (PBS) and made up to 10 mL with ultra high quality (UHQ) water. Sections were then incubated in primary antibody, either anti-Sjcytb561 or anti-KLH, for 30 minutes. Primary antibodies were diluted 1∶75 in blocking buffer and the negative control was blocking buffer only. Grids were washed in blocking buffer for 4×5 min and incubated in Protein A conjugated to 10 nm colloidal gold particles (Aurion Immuno Gold Reagents & Accessories) for 30 minutes at a 1∶100 dilution. Subsequently, sections were rinsed with 4×5 minute washes in PBS and 4×2 minutes washes in UHQ water. Sections were contrasted using uranyl acetate (1 minute) and lead citrate (30 seconds) and viewed using a JEM 1011 transmission electron microscope operating at 80 kv and equipped with digital camera system.

### Functional expression of Sjcytb561 in yeast

Yeast strains S288C wildtype (MATα ura3-52 leu2-1) and mutant S288CΔfre1Δfre2 (MATα fre1:URA3 fre2:HISG leu2-1) used in the assays were a gift from Professor Jerry Kaplan (University of Utah, USA). Full-length *Sjcytb561* cDNA was amplified by PCR from adult worms and cloned into the pESC-Leu vector (Stratagene). Yeast genomic DNA was obtained from the S288C wildtype strain using Prepman Ultra Sample Preparation Reagent (Applied Biosystems) according to the manufacturer's instructions. The resulting template was used to amplify the *Saccharomyces cerevisiae fre1* gene (GenBank accession no. M86908), which was cloned into pESC-Leu as a positive control. The clones were transformed into the S288CΔfre1Δfre2 cells according to the manufacturer's (Stratagene) protocol. The transformed yeast cells were grown in SD dropout medium lacking Leucine (SD-Leu). Expression was induced by transferring to dropout medium containing galactose rather than dextrose (SG-Leu).

Ferrireductase assays were carried out as described [28]. Briefly, for the plate assay, low iron plates were made (SG-Leu minimal medium, 2% (w/v) agarose, 50 µM Fe^3+^- ethylenediaminetetraacetic acid (EDTA). Fifty µM bathophenanthroline disulphonate, (BPS, Sigma), a ferrous iron chelator, was added to remove any residual ferrous iron from the plates. Transformed yeast were grown overnight in liquid culture medium. Cells were grown until the culture reached an OD_600_ of 0.8. Cells were then centrifuged, washed twice with double distilled water. Five µL of cells were plated at different concentrations and grown for 3 days at 30°C. For the cell-surface ferrireductase assays, cells were grown, collected and washed as per the plate assays, but were resuspended in assay buffer (5% (w/v) glucose, 0.05 M sodium citrate, pH 6.5). For the ferricyanide (FeCN) reductase assays, cells were added to assay buffer, with FeCN to a final concentration of 0.356 mM. The absorbance change was monitored for 5 min at 420 nm and the ferricyanide reduction rates were calculated using the established extinction coefficient of 1.02 mM^−1^cm^−1^ [28]. For Fe^3+^-EDTA reductase assays, cells were added to assay buffer containing 1 mM ferrozine and 250 µM Fe^3+^-EDTA as substrate. Cells were incubated with shaking at 30°C. Over a time course, 1 mL of cells was removed, centrifuged for 20 seconds and the absorbance of the supernatant measured at 562 nm. Blanks containing assay buffer and ferrozine, but no cells, were incubated at the same time, and the corresponding readings were subtracted from each sample time point. The previously established extinction co-efficient for the formation of ferrozine-ferrous iron complex of 27.9 mM^−1^cm^−1^ was used in the activity calculations [28].

## Results

### Identification of a cytochrome b561 in *S. japonicum*

**Figure 1:**
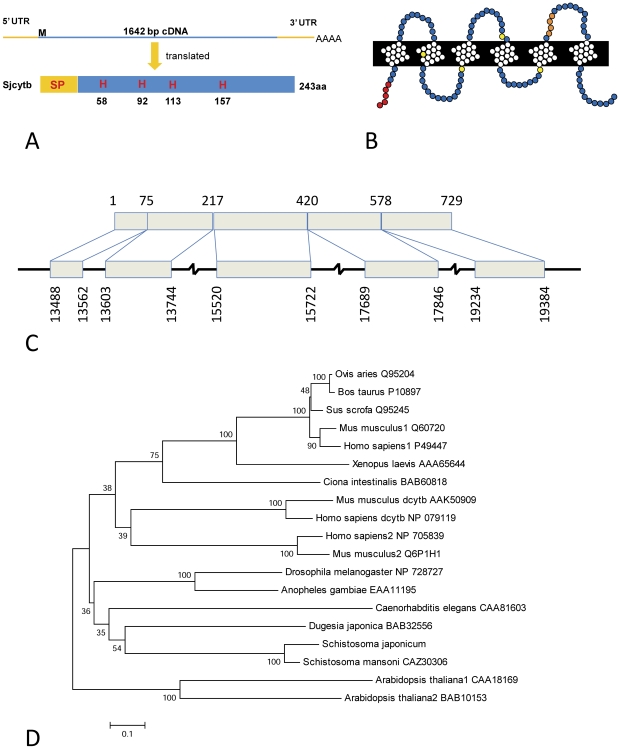
Bioinformatic and phylogenetic sequence analysis of SjCytb561. A. Schematic diagram of the SjCytb561 cDNA and protein sequence. The protein sequence contains a predicted signal peptide (SP) and the four conserved histidine residues (H) shown in red. B. Schematic representation of the predicted membrane topology and orientation of full length SjCytb561. The 5′ N-terminal signal peptide sequence is shown in red and the conserved histidine residues that bind two heme molecules and confer tertiary structure are coloured yellow. The region depicted in orange indicates the location of the peptide sequence that was used for antibody production. C. Genomic organization of *Sjcytb561*. The cDNA structure is presented at the top with exon sequence positions shown. Below is the corresponding cDNA structure with exons shown as boxes and intermediate intron regions shown as solid lines; again relative sequence positions are noted. D. Minimum evolution phylogenetic tree inferred from amino acid sequences of cytochrome b561 genes from a variety of organisms. Species name and published accession numbers are shown for each sequence. Bootstrap values (1000 resamplings) are shown to the left of the relevant nodes.

*Schistosoma japonicum* datasets were searched by BLAST to identify homologues of known ferric reductase proteins from other organisms. This search revealed an EST with homology to the cytochrome b561 family of proteins (Genbank, AY816000). Using RACE PCR, the full-length cDNA was obtained, sequenced and revealed a 1645 bp coding region. The translated sequence encoded a 243 amino acid protein (Fig. 1A–B), with a predicted molecular mass of 26.9 kDa.

### *In silico* analysis of the Sjcytb561 sequence

The recent release of the *S. japonicum* genome [31] allowed us to compare the sequence we confirmed for Sjcytb561 with the putative *in silico* predicted sequence from the genome project. Using BLAST function against the sequences in the *S. japonicum* genome project (GeneDB_Sjaponicum_Gene.v4.0), we identified the region of the genome in which the gene is located. BLAST analysis of the genomic contigs (sjr2_contig.fas) identified a region designated as: >gnl|lsbi|CNUS0000126016.1 (*Schistosoma japonicum* isolate Anhui (wildtype) SJC_C017791, Length  = 24880, Bit Score  = 402 E value = e-110, Identities  = 203/203 (100%)) as the genomic site of *Sjcytb561*. Using the program Sim 4, we aligned cDNA sequence to the contiguous genomic sequence previously identified by BLAST. This resulted in the identification of 5 exons (Fig. 1C).

**Figure 2:**
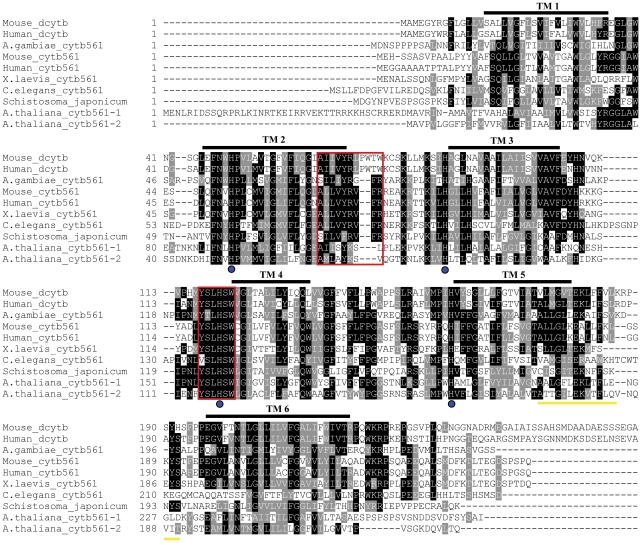
Multiple sequence alignment (ClustalW) of the deduced amino acid sequence of *SjCytb561* with sequences in other taxa belonging to the Cytb561 family. Sequences included are: Human (GenBank accession no: NP_079119) and mouse (AAK50909) Dcytb, and other Cytb561s from human (P49447), mouse (Q60720), *Anopheles gambiae* (EEA11195), *Xenopus laevis* (AAA65644), *Caenorhabditis elegans* (CAA81603) and *Arabidopsis thaliana* 1 (CAA18169) and 2 (BAB10153). Putative transmembrane regions are spanned by a black line and labelled TM. The conserved histidine residues are indicated by the blue circles and the substrate-binding regions are shown in red boxes. The *S. japonicum* sequence used for the construction of a synthetic peptide for antiserum production is shown underlined in yellow.

Analysis of the translated nucleotide sequence revealed the presence of a signal peptide. The predicted topology of the putative protein contained six transmembrane spanning regions, four of which formed the ‘core domain’ identifiable in the cytochrome b561 family of proteins [15]. In addition to these six transmembrane helices, the predicted peptide of *S. japonicum* contains four completely conserved histidine residues (thought to co-ordinate two heme centers) (Fig. 2) and predicted substrate binding sites (Fig. 2). Despite these completely conserved structural features, the cytb561 sequences exhibit relatively low homology both within and among species (Fig. 2). The *S. japonicum* sequence shared between 30 and 45% homology with other cytb561 family members (identity with human Dcytb, NP_079119 was 38%; with that of *Arabidopsis* [GenBank accession no. CAA18169], 31%; and with *Xenopus cytb561* [AAA65644], 44%). A homologous sequence (Smp 136400) was also identified in the *S. mansoni* GeneDB, which is a first pass annotation of the *S. mansoni* genome assembly, with 85% identity to the *S. japonicum* sequence (http://www.genedb.org/genedb/smansoni/).

Minimum evolution phylogenetic analysis (Fig. 1D) showed the schistosome sequence grouped most closely with that of the freeliving turbellarian, *Dugesia japonica*, another member of the Phylum Platyhelminthes. However, this grouping was not supported, having a low bootstrap value. There was a distinct clustering of mammalian duodenal cytochromes (Human, NP_079119 and Mouse AAK50909) relative to other mammalian cytb561 proteins. There are also distinct branches representing the type 1 and type 2 mammalian cytochromes b561. Type 1 cytb561 members include neuroendocrine expressed proteins, such as chromaffin granule cytochromes, which are present in the adrenal medulla (Human, P49447, Mouse, Q60720). Type 2 mammalian cytsb561, found in humans and the mouse, are termed ‘ubiquitious’ cytochromes as they are more widely expressed (Human, NP_705839, Mouse, Q6P1H1) [24]. Individual functions of the proteins contained in this tree have not all been experimentally assessed and confirmed, particularly those from invertebrates.

### Stage specific expression of Sjcytb561

**Figure 3:**
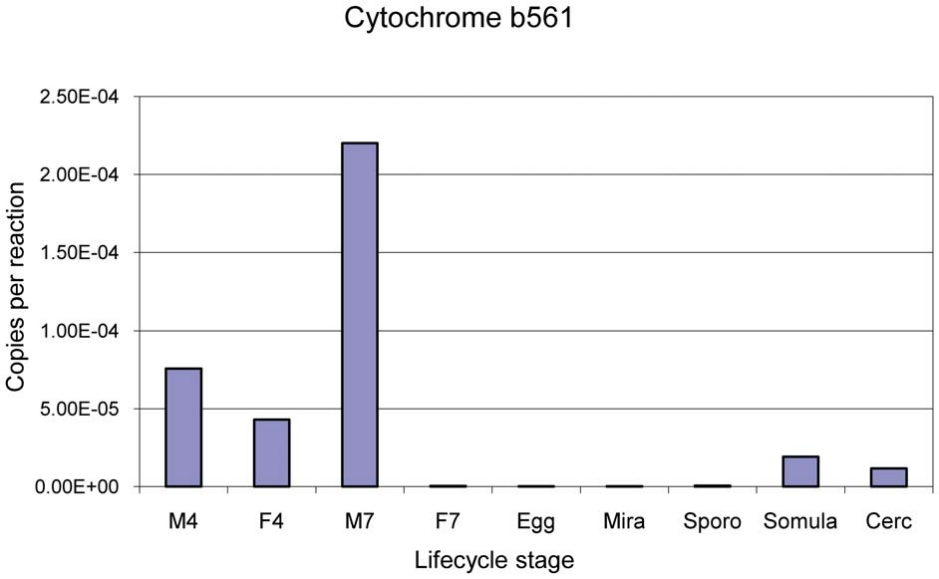
Expression of *SjCytb561* in different *S. japonicum* life cycle stages. Abbreviations: M4, male 4 weeks post infection (wpi); F4, females 4 wpi; M7, male 7 wpi. F7, females, 7 wpi; Egg, eggs; Mira, miracidia; Sporo, sporocyts; Somula, - lung-stage schistosomula; Cerc, cercariae.

Quantitative analyses of expression of SjCytb561 in different stages of the life cycle (Fig. 3), showed highest mRNA expression in adult (7-week-old) males, with less expression in developing (4-week-old) males and females. Expression in the 7-week-old females appeared to diminish compared with 4-week-old females. Lower expression levels were evident in the schistosomula and cercariae.

### Western blot analysis and immunolocalisation of native Sjcytb561

**Figure 4:**
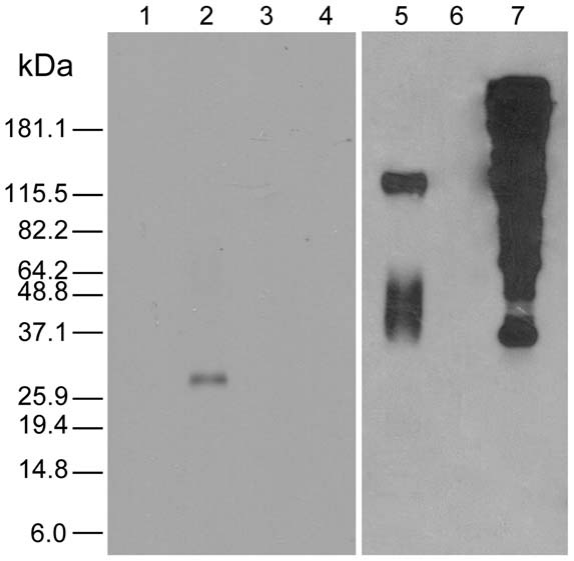
Western blot analysis of *S. japonicum* Cytb561. Lanes 1 to 3 were probed with, anti-SjCytb561 serum. Lane 1 probed against an adult worm ‘stripped’ extract shows no reactivity. Lane 2, probed against adult tegument extract shows a band at approximately 30 kDa. Lane 3 was probed against 5 µg of commercial KLH protein, and shows no reactivity. Lane 4 is pre-immune serum probed against adult worm tegumental extract and shows no reactivity. Lanes 5 to 7 were probed with anti-KLH antibodies. Lane 5 is against ‘stripped’ worm extract and shows a number of bands. Lane 6 shows tegument extract and no reactivity. Lane 7 contained 5 µg of KLH and shows intense reactivity against the anti-KLH sera.

Western blot analysis (Fig. 4) of adult schistosome membrane extracts using KLH-depleted anti-Sjcytb561 sera showed a tight band at approximately 30 kDa (Fig. 4, lane 2), which is in accordance with the predicted molecular mass of 26.8 kDa. No band was observed with pre-immune sera probed against the tegument exacts (Fig. 4, lane 4). Anti-SjCytb561 sera probed against commercial KLH did not elicit a reaction, suggesting KLH-cross-reactivity was not a problem (Fig. 4, lane 3). Further, anti-KLH antibodies did not show reactivity against the schistosome tegument extracts (Fig. 4, lane 6).

**Figure 5:**
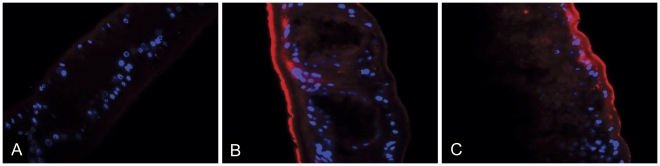
Immunolocalisation of SjCytb561 in *S. japonicum* male adult worms using primary rabbit sera and secondary sera conjugated to Cy3, and DAPI counterstain. Panel A shows an adult male worm probed with pre-immune sera. Panels B and C show reactivity over the tegument and basally to the tegumental matrix after localisation with anti-SjCytb561 serum.

Sections of adult worms probed with pre-immune rabbit serum and secondary serum conjugated to Cy3 did not show any significant fluorescence (Fig. 5A), nor did the secondary antibody only sections (not shown). Sections probed with anti-Sjcytb561 antibodies showed strong labelling on the distal cytoplasm of the outer tegument (Fig. 5B and 5C) and more diffuse reactivity over the underlying parenchyma. These results suggest a tegumental role for cytochrome b561 in *S. japonicum*.

**Figure 6:**
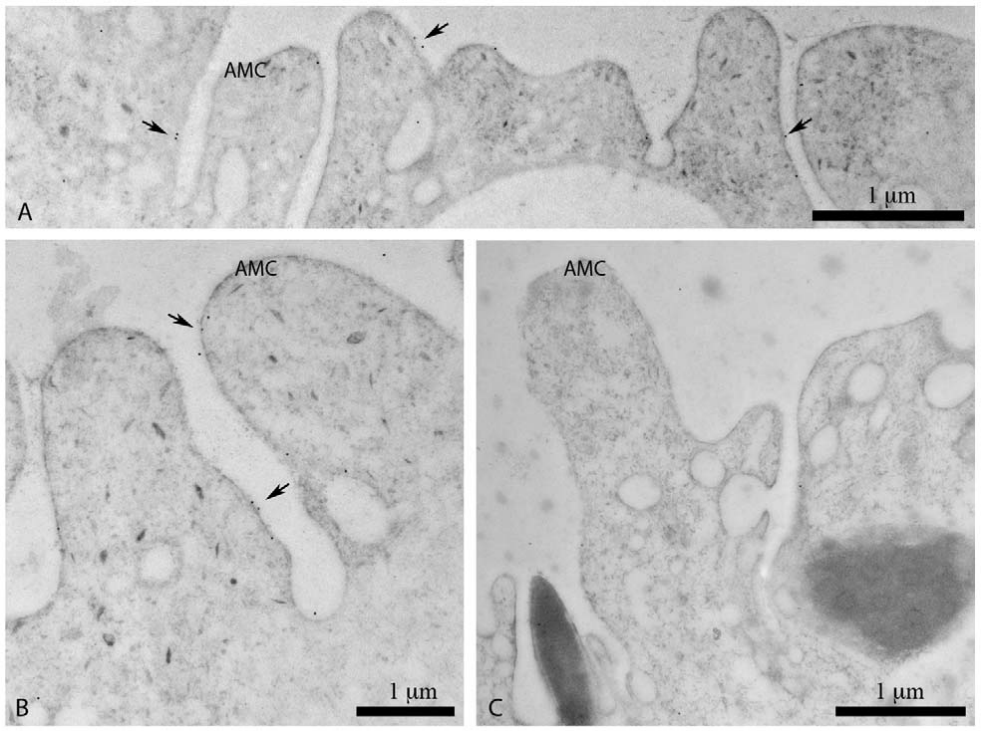
Electron micrographs of *S. japonicum* male adult worm tegument showing immunogold labelling of SjCytb561 and KLH. Panels A and B were labelled with anti-SjCytb561 sera and show gold probes (examples indicated by arrows) over the apical membrane complex (AMC) of the tegument. Panel C was labelled using anti-KLH sera and does not show labelling in the AMC.

Electron micrographs of adult tegument probed with anti-SjCytb561 serum (Fig. 6A and 6B) showed the presence of gold probes in the apical membrane complex. Labelling with anti-KLH serum and with the negative controls did not show labelling of gold probes in this region.

### Functional expression of Sjcytb561 in a yeast mutant model

**Figure 7:**
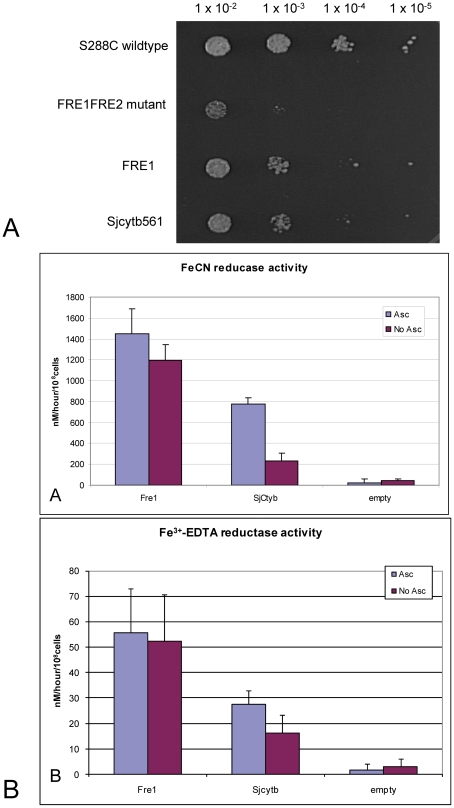
SjCytb561 can rescue the growth defect of Δfre1Δfre2 mutant yeast cells. A. Growth on solid phase media. Five microlitres of cells at different concentrations were plated on low iron media containing 50 µM Fe3+-EDTA as iron substrate. Fifty µM BPS (iron chelator) was also added to remove any free ferrous iron. Empty vector was transformed into wildtype and mutant cells to enable growth of SG-Leu media. B. Ferric reductase activity of SjCytb561 in intact yeast cells. Vectors were transformed into Δfre1Δfre2 mutant cells to measure ferric reductase activity using FeCN (A) and Fe3+-EDTA (B) as ferric iron substrates, in the presence or absence of ascorbate (ASC).

In light of the discovery that some cytochromes b651s exhibit ferric reductase capability [26], [27], [28], [29], [40], the *S. japonicum* cytochrome b561 was expressed in a yeast model deficient in ferric reductase activity. Fre1 and Fre2 are yeast ferrireductases that are responsible for almost all ferric reductase activity at the plasma membrane [6], [41], [42]. The fre1Δfre2Δ mutant yeast grows poorly in iron-restricted conditions, but can be rescued by complementation with the Fre1 gene. The Sjcytb561 protein was expressed in this mutant model to determine whether it exhibits ferric reductase activity. Empty vector was used as the negative control in these experiments. Growth of the transformed yeast on solid phase media showed that the presence of the Sjcytb561 protein restored growth equal to that of the Fre1 positive control, indicating the molecule has ferric-reductase activity (Fig. 7A).

To further investigate the kinetics of ferric reductase activity occurring in the rescued cells, assays were performed on intact yeast cells in liquid culture (Fig. 7B). Ascorbate is the predicted and commonly accepted electron donor for cyts-b561 under physiological conditions [43], [44], so cultures were also tested in the presence or absence of ascorbate to determine its contribution to activity. The cells transformed with empty vector did not exhibit significant ferric reductase activity. The Fre1-transformed cells had a high level of ferric reductase activity that was independent of the presence or absence of ascorbate. The Sjcytb561 transformed cells also exhibited a high level of ferric reductase activity, which was greatly enhanced by the addition of ascorbate. There was also a difference seen in the form of iron substrates used in the experiments, with FeCN a more efficient substrate.

## Discussion

All organisms that require iron have at least one ferrous iron transport system, and often many more than one. The limited availability of ferrous iron in many environments has necessitated the evolution of a range of ferric reductases to allow ferrous iron uptake to occur [2], [8]. Cytochrome b561 has been identified in a large number of phylogenetically distant taxa and many species contain more than one cytb561 [24]. This family of proteins has long been known to play a role in ascorbate regeneration and electron transfer, but its role as a reducer of ferric iron has only been recognised recently [26], [27], [28], [29], largely through studies of mammalian duodenal cytochrome b [10], [27], [40], [45], [46].

Sjcytb561 is the first of this family of proteins identified from any parasitic platyhelminth or nematode, which is surprising as the protein is found ubiquitously in the plant and animal kingdoms [24]. The *S. japonicum* cytb561 has all the structural hallmarks of the family, including the six transmembrane spanning regions, four completely conserved histidine residues and the conserved substrate binding sites (Fig. 2). However, unlike other organisms which possess multiple Cytsb561, *S. japonicum* and *S. mansoni* appear to have only one Cytb561 transcript in their respective genomes. A study by Tsubaki and colleagues [15] aimed to predict the function of all known members of the cytochrome b561 family based on protein sequence motif analyses in the conserved binding regions. This study predicted that only a subset of the family would have ferric reductase function. Notably, the Sjcytb561 protein identified here was predicted to belong to the group with ferric reductase potential [15]. The yeast rescue assay presented here provides the first evidence of ferric reductase activity in a non-mammalian metazoan cytochrome b561.

Yeast is one of the best characterised model eukaryotes for iron metabolism and iron transport. Fre1 and Fre2 are responsible for almost all of the ferric reductase activity in yeast cells that if knocked out result in a significant defect in growth [3], [6], [41], [42], [47],[48],[49]. Functional assays in the yeast Δfre1Δfre2 mutant demonstrated the ability of Sjcytb561 to reduce ferrous iron and, thereby, restore iron uptake and normal growth (Fig. 7). This mechanism for the *S. japonicum* Cytb561 appears to be ascorbate-dependent, as there was a significant increase in reductase activity with the addition of ascorbate. Fre1 transformed yeast are not affected in the same manner by the addition of ascorbate, as Fre1 is a NADPH-dependent reductase [41]. Yeast are unable to synthesise ascorbate but they do synthesise a homologue called erythroascorbate. The low ferric reductase activity that was observed without the addition of ascorbate may suggest that erythroascorbate, or some other reductant, in the yeast cells may be able to function as the electron donor in this reaction [43], [44]. The ability to synthesise ascorbate has been lost a number of times in evolution, most notably in humans, and it is unknown if schistosomes are able to synthesise their own ascorbate or, if indeed, it too must be scavenged from the host [50], [51].

The localisation of Sjcytb561 to the tegument (Fig. 5 and 6) is consistent with that of schistosome DMT1, a known ferrous iron transporter [23]. It is possible that these two proteins act together to take up iron from the host, although the presence of other ferric reductases at this site cannot be excluded. Indeed, the lack of phenotype observed in mammalian Dcytb knockout studies makes it likely that there are other ferric reductases able to perform this role [54]. Neither the schistosome DMT1 nor this newly identified cytochrome b561 have been identified in proteomic studies of the schistosome tegument [39], [52], [53]. Iron homeostasis proteins of eukaryotes show transcriptional changes in response to cellular iron levels, whereby expression of proteins involved in acquisition and utilization of iron are increased in response to iron deficiency [54]. Mechanisms also exist to change the sub-cellular localisation of iron transport proteins, a process that is considered the ‘first line’ response of cells after changes in iron levels. It is often necessary to induce iron-starvation conditions to achieve significant up-regulation for the analysis of these proteins [54]. Given that the previous proteomic studies [39], [52], [53] were conducted on parasites obtained from iron-replete hosts it is possible that the schistosome iron transport molecules are, in fact, present at very low levels and combined with changes in sub-cellular localisation were not identified in these analyses. Future proteomic studies could be conducted on parasites cultured under iron-deficient conditions to confirm this. Mammalian duodenal cytochrome b has been demonstrated to reduce copper *in vitro* and it is possible that cytochromes b561 may play a more general role in metal iron uptake [55]. The molecular mass of iron is similar to metals such as copper, manganese, zinc and cobalt and most other ferrous iron transport systems can also take up these and other transition metals [2]. Notably, a transporter for copper has been identified in proteomic studies of schistosome tegument extracts [52].

The high expression levels for *Sjcytb561* in adult males complemented the tegument localisation data. *In vivo*, the male worm surrounds the female schistosome, holding her in his gynaecophoral canal. The tegument of the male is in constant contact with host serum molecules, and has a larger surface area than that of the female [56]. Hence, Sjcytb561 would be expected to be highly expressed at this site in adult males. The reduction of transcript expression from the 4-week females to the 7-week females is logical since at 7 weeks more genes would be upregulated for egg production, rather than purely for growth and nutrition needs. The question of iron utilisation in egg production by females, be it for egg shell formation or for use by the developing miracidia within the egg [17], [18], remains undefined. However, the relatively low SjCytb561 ferric reductase expression levels in the miracidia and eggs of *S. japonicum* is noteworthy in that it suggests that if iron is being liberated from vitelline cells by the developing embryo, it is unlikely to involve cytochrome b561 reductase-mediated release of iron.

It has been postulated that ferric reductases, by decreasing the affinity of biological carriers for iron, may play a role in the removal of iron from chelators [57]. In the case of schistosomes, this may mean aiding in the release of iron from the abundant host serum iron glycoprotein, transferrin. It has also been hypothesised that using ferric reductase coupled with iron uptake mechanisms may allow parasites and other pathogens to survive in a more diverse range of hosts and tissues, than organisms restricted to specific receptor-mediated iron uptake [13] [58]. In the absence of any clear research regarding haem uptake and usage in schistosomes it can be postulated that this surface mediated iron uptake mechanism is at least used to supplement the adult worms iron needs.

*S. japonicum* cytb561 is the first ferric reductase identified in any parasitic helminth and emphasises the importance of iron and other divalent cations in this group of important parasites. Further understanding of the mechanisms of metal uptake and utilization in schistosomes may uncover novel drug and vaccine targets for controlling schistosomiasis.
